# Stabilizing Layered Cathodes by High-Entropy Doping

**DOI:** 10.34133/research.0503

**Published:** 2024-10-23

**Authors:** Yuan Ma, Zihao Zhou, Torsten Brezesinski, Yanjiao Ma, Yuping Wu

**Affiliations:** ^1^ Confucius Energy Storage Lab, School of Energy and Environment, Southeast University, Nanjing 211189, China.; ^2^ School of Energy and Mechanical Engineering, Nanjing Normal University, Nanjing 210023, China.; ^3^ Institute of Nanotechnology, Karlsruhe Institute of Technology, 76131 Karlsruhe, Germany.

## Abstract

Layered Ni-rich oxide cathodes in lithium-ion batteries (LIBs) often struggle with poor thermal safety and capacity fade. Xin and colleagues’ studies in *Nature* and *Nature Energy* demonstrate a novel high-entropy (compositionally complex) doping strategy, introducing “cocktail effects” from multiple constituents. This approach substantially improves cycling performance and stability, reduces material cost, and may pave the way toward the development of advanced electrodes for next-generation LIBs.

Unlike traditional doping (or substitution) strategies, high-entropy doping is characterized by simultaneously introducing a variety of principal elements, typically 5 or more [1]. Materials modified using this approach often exhibit enhanced redox and/or catalytic activity. However, the improvements cannot be attributed to specific attributes of a single component (dopant), but instead originate from the unique interactions among the different elements, sometimes leading to unexpected features of the system [2]. By tailoring the element combinations and stoichiometric ratios, the interactions can be altered to achieve superior performance in practical applications [3–7]. Two recent studies by Xin and colleagues, published in *Nature* [8] and *Nature Energy* [9], demonstrate the substantial potential of high-entropy or, more properly, compositionally complex doping strategies for improving the cyclability of layered Ni-rich oxide cathode active materials (CAMs) in lithium-ion batteries (LIBs), as shown in the Figure.

**Figure.**
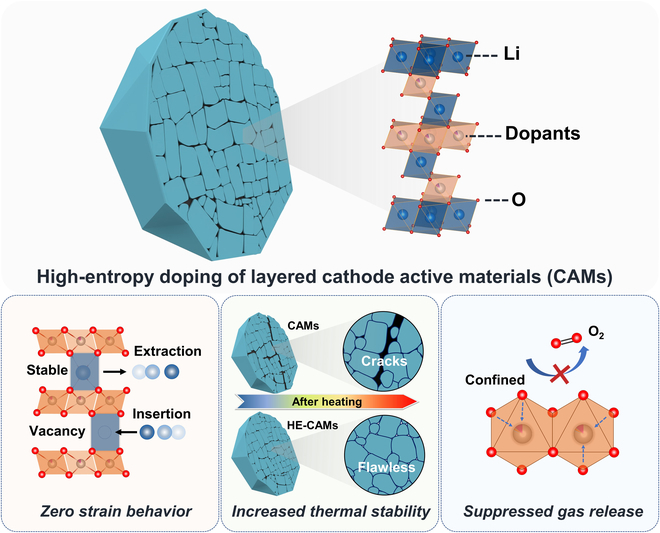
Schematic of the high-entropy doping strategy for addressing challenges in layered oxide cathodes for battery application.

Owing to the high theoretical specific capacity (*q*_th_ ≈ 275 mAh/g) and high mean discharge voltage (≥3.7 V versus Li^+^/Li), Ni-rich cathodes like LiNi_0.8_Co_0.1_Mn_0.1_O_2_ (NCM-811) are of great academic and industrial interest. However, the intrinsically poor thermal tolerance of these CAMs (due to the strong oxidizing nature of Ni^4+^ and lattice oxygen release, especially at high states of charge) and chemo-mechanical stability issues (caused by adverse phase transitions and cracking due to strain localization) pose a significant safety concern and further lead to performance decay, which in turn impedes their commercialization [10,11]. To address these problems, Xin and colleagues successfully applied the high-entropy doping strategy to NCM-811 and LiNi_0.5_Co_0.3_Mn_0.2_O_2_ (NCM-532). In doing so, they were able to achieve major improvements in energy density and cycling stability. Aside from that, it also allowed for complete replacement of Co in both CAMs, thus effectively reducing material cost and, at the same time, addressing ethical issues.

In the first paper published in *Nature* [8], the authors reported on a new Co-free CAM, namely, LiNi_0.8_Mn_0.13_Ti_0.02_Mg_0.02_Nb_0.01_Mo_0.02_O_2_ (referred to as HE-LNMO). The synthesized HE-LNMO exhibited a well-defined layered structure with uniformly distributed dopants, except for Ti, Mo, and Nb, the latter being slightly enriched on the primary particle surface. Previous studies have demonstrated that the key to improved performance lies in the synergistic effect of all constituents (so-called “cocktail effects”). In particular, doping strategies involving various species simultaneously offer the potential to establish a stable CAM lattice structure and to reduce the strain accumulation occurring upon lithium insertion/extraction. HE-LNMO has been shown to undergo minor volume variations of about 0.3% (quasi-zero stain behavior) during battery operation, compared to 2.7% for NCM-811. This effectively mitigates mechanical degradation (fracture) of the CAM particles. In addition, multicomponent doping may also help to suppress loss of lattice oxygen under harsh conditions (high temperature), apparently due to immobilization of oxygen vacancies surrounding the high-valence dopants (Ti, Nb, and Mo). Because of these advantages, HE-LNMO exhibited superior electrochemical performance. For example, the initial Coulomb efficiency at C/10 rate was much higher than that of cells using NCM-811 (94% versus 82%), although both CAMs delivered similar initial specific discharge capacities of about 210 mAh/g. In the subsequent cycling, HE-LNMO showed promising behavior with a capacity retention of 98.5% after 100 cycles in the potential range of 2.5 to 4.4 V versus Li^+^/Li, clearly surpassing that of NCM-811 (87.1%). By increasing the upper cutoff potential to 4.5 V, the capacity retention of the HE-LNMO cells (after 50 cycles) remained around 98%, whereas NCM-811 underwent rapid fading, achieving only 85.8% capacity retention. It should be noted that both electrochemical testing of single-layer pouch cells with a graphite anode and high-temperature cycling provided additional evidence of the suitability of HE-LNMO for industrial use. Notably, the HE-LNMO-based full cells revealed a capacity retention of about 95% after 500 cycles (2.8 to 4.2 V), similar to that achieved with state-of-the-art Ni-rich CAMs.

By decreasing the Ni content while maintaining a moderate Co content in layered CAMs, their cycling performance can be improved to some extent [12]. Hence, it appears likely that NCM-532 will continue to have some share of the cathode market in the next years. Nevertheless, removing Co from NCM-532, aiming at low-cost LIBs, without sacrificing performance remains challenging [13,14]. Writing in *Nature Energy* [9], Xin’s group again applied the high-entropy concept to design a new medium-Ni and Co-free CAM, namely, LiNi_0.5_Mn_0.43_Ti_0.02_Mg_0.02_Nb_0.01_​Mo_0.02_​O_2​_ (referred to as HE-N50), which further confirms the feasibility of combining Mn, Ti, Mg, Nb, and Mo as dopants for optimizing LIB cathodes. Similarly, the different elements were found to be uniformly dispersed in the bulk, with minor variations in concentration for the high-valence dopants on the HE-N50 particle surface. In addition to the layered phase (*R−*3*m* space group), about 5% of Li_2_MnO_3_ crystallizing in the *C*2/*m* space group was also detected. The formation of this impurity phase has been associated with some localized excess of lithium and the relatively high Mn content.

Overall, the findings indicate that the inclusion of high-valence transition metal species triggers the activation of the Mn^3+^/Mn^4+^ redox couple during cycling. Furthermore, the even distribution of Mg in the primary particles helped to mitigate Li^+^/Ni^2+^ mixing or, in other words, the formation of substitutional defects (${\mathrm{Ni}}_{\mathrm{Li}}^{•}$), which has a beneficial effect on the cyclable lithium inventory. In fact, HE-N50 outperformed NCM-532 after 20 cycles at C/3 rate (2.7 to 4.5 V versus Li^+^/Li). The compositionally complex doping was again found to reduce electrode breathing (volumetric changes) during delithiation and relithiation. As a result, the HE-N50 cells delivered a high specific energy of 642 Wh/kg with 95% capacity retention after 100 cycles, compared to 564 Wh/kg and 83% for NCM-532. In pouch-type full cell testing, HE-N50 showed much superior stability, retaining almost 95% of the initial capacity after 1,000 cycles at 1C rate (2.8 to 4.3 V).

This kind of doping strategy also shows promise in increasing the thermal stability of layered Ni-rich oxide CAMs. The NCM-532 particles exhibited numerous cracks, as well as the formation of disordered spinel and rock-salt domains upon heating the delithiated material at 350 °C for 30 min. In contrast, HE-N50 underwent phase transformation from layered to ordered spinel without any visible cracking. This result suggests that the latter CAM has better thermal stability than NCM-532 and shows more resistance to oxygen loss.

Recent studies have conducted a deeper exploration of high-entropy doping strategies, considering more nuanced factors in element selection, such as the impact of electronegativity. For instance, Liang et al. found that selecting low electronegativity cationic high-entropy dopants can activate the reversible redox activity of anions in layered Ni-rich cathodes, thereby achieving additional capacity and maintaining structural stability [15]. Additionally, high-entropy doping strategies can be integrated with other approaches, such as single-crystal synthesis. This integration, for example, has led to the development of Co-free Ni-rich CAM, which exhibits unprecedented cycling stability in both half and full cells [16]. Taken together, compositionally complex doping or substitution strategies may pave the way toward the development of next-generation electrode materials for application in LIBs with unprecedented energy density and longevity. Furthermore, they seem to diversify the range of elements that can be considered for modification, with cocktail and other effects determining the overall performance, eventually overcoming limitations of traditional approaches. Overall, high-entropy doping is poised to become a key cornerstone in the future of materials science.
